# Impact of Downregulated Inflammation via the *IL6R* Asp358Ala Variant on Atrial Fibrillation Recurrence Following Ablation

**DOI:** 10.1016/j.jacadv.2026.103285

**Published:** 2026-09-23

**Authors:** Jitae A. Kim, Jisong J. Kim, Caique M.P. Ternes, Mihail G. Chelu, Anne B. Curtis

**Affiliations:** a Division of Cardiovascular Medicine, Jacobs School of Medicine and Biomedical Sciences, University at Buffalo, Buffalo, New York, USA; b Joe R. and Teresa Lozano Long School of Medicine, San Antonio, Texas, USA; c Department of Medicine, Section of Cardiology, Baylor College of Medicine, Houston, Texas, USA; d Cardiovascular Research Institute, Baylor College of Medicine, Houston, Texas, USA; e Texas Heart Institute at Baylor College of Medicine and Baylor St. Luke’s Medical Center, Houston, Texas, USA

**Keywords:** ablation, atrial fibrillation, inflammation, interleukin-6, rhythm control

## Abstract

**Background:**

Elevated interleukin (IL)-6 levels are associated with the development of atrial fibrillation (AF). Whether downregulation of IL-6 mediated inflammation reduces AF recurrence following ablation is uncertain.

**Objectives:**

Using a common variant in the IL-6 receptor gene, *IL6R* Asp358Ala, as a genetic proxy for IL-6 receptor blockade, we evaluated the effect of impaired IL-6 signaling on AF recurrence following ablation.

**Methods:**

We identified patients undergoing AF ablation with genomic data in the All of Us Research database. Patients were stratified based on the presence of the *IL6R* Asp358Ala allele. The primary outcome was AF recurrence, defined as a composite of repeat AF ablation, cardioversion, or new class I/III antiarrhythmic drug, following a 3-month blanking period after ablation.

**Results:**

Of the 1,540 patients undergoing AF ablation, 707 patients carried 1 copy of the Asp358Ala variant and 226 carried 2 copies. At a median follow-up of 1,317 days, the AF recurrence rate was 14.2 per 100 patient-years for carriers of Asp358Ala as compared to 15.9 for noncarriers (adjusted HR: 0.93, 95% CI: 0.79-1.10). No difference in recurrence was observed when using an additive model (2 vs 1 vs no variant allele), suggesting no dose-response with the variant. In subgroup analysis of patients with heart failure (n = 312), there was a potential beneficial effect of Asp358Ala on recurrence (adjusted HR: 0.72, 95% CI: 0.52-1.00; interaction *P* = 0.049).

**Conclusions:**

Targeted IL-6 inhibition may have little impact on long-term AF recurrence in the general AF population undergoing ablation, although there may be potential benefit in heart failure patients.

Atrial fibrillation (AF) is the most common sustained arrhythmia, with a lifetime risk of 1 in 3 to 5 individuals aged above 45.^1^ Catheter ablation is an effective treatment for drug-refractory AF and is increasingly utilized as first line therapy in select patients.^2^^,^^3^ However, despite advances in ablation technologies and techniques, recurrence rates following AF ablation remain substantial, often necessitating repeat ablation procedures.^4^

Although the pathogenesis of AF is multifactorial, including genetic and clinical risk factors, systemic inflammation has been increasingly recognized as an independent risk factor for the development of AF through atrial structural and electrical remodeling.^5^, ^6^, ^7^, ^8^, ^9^ Given its role in the pathophysiology of AF, systemic inflammation may also play a role in AF recurrence following ablation.^10^ Among several biomarkers of inflammation, interleukin (IL)-6, a potent proinflammatory cytokine, is suggested to have the strongest association with AF susceptibility.^8^^,^^11^, ^12^, ^13^ IL-6 has been shown to directly contribute to AF in animal models, further supporting a preeminent mechanistic role for IL-6 in AF.^14^ However, whether IL-6 represents a therapeutic target to mitigate AF recurrence after ablation remains uncertain.^10^

A common missense variant (allele frequency 39%) in the IL-6 receptor (IL6R) gene, *IL6R* Asp358Ala (rs2228145 A > C), has been frequently utilized as a genetic proxy for evaluating the effects of IL-6 downregulation on a variety of cardiovascular diseases.^15^, ^16^, ^17^, ^18^, ^19^ The variant leads to reduced cell surface expression of IL6R and attenuated classical IL-6 signaling, analogous to IL6R blockade with tocilizumab.^16^^,^^20^ *IL6R* Asp358Ala is associated with a decreased incidence of AF,^12^^,^^21^^,^^22^ although its effects on long-term AF ablation outcomes have not been well evaluated. Therefore, we sought to explore whether genetically proxied IL-6 downregulation with the *IL6R* Asp358Ala variant could be associated with decreased AF recurrence following ablation.

## Methods

### Study population and data source

This retrospective cohort study was performed using data from the All of Us Research Program Controlled Tier Data Set version 8 (C2024Q3R9). The All of Us Research Program is an National Institutes of Health–funded research initiative that seeks to create a diverse U.S.-based cohort biobank to accelerate precision health research. The All of Us Research Program currently includes data from more than 633,000 participants across >340 sites, with the goal of enrolling a diverse group of at least 1 million persons in the United States. Whole genome sequencing is available from more than 414,000 participants, with more than 80% of participants from groups that have been historically underrepresented. Participant data in the All of Us Research Program also include electronic health record (EHR) data, health surveys, digital health data, and physical measurements collected at enrollment. EHR data are abstracted using the Observational Medical Outcomes Partnership Common Data Model to harmonize clinical data across various health care systems and includes diagnoses, visits, procedures, medications, and laboratory values. The All of Us Research Program protocol has been previously described in detail.^23^ All of Us data are publicly available through the Researcher Workbench, with access granted to researchers at participating institutions following training in the ethics of research involving the All of Us data (https://allofus.nih.gov/). As only publicly available deidentified data were used in the present study, Institutional Review Board and prior ethics approval were not required.

### Study population identification

All patients in the All of Us data set undergoing a first time AF ablation procedure, identified by the Current Procedural Terminology code 93656, with available whole genome sequencing data were included in the present study.

### Outcomes

The primary outcome was AF recurrence following a standard 90-day blanking period after the index ablation procedure. AF recurrence was defined as the composite of repeat AF ablation procedures, cardioversion (CV), or new class IC or III antiarrhythmic drug (AAD) usage. CV was identified using the Current Procedural Terminology code 92960, whereas AADs were identified using their corresponding standard concept names in the Observational Medical Outcomes Partnership Common Data Model. Secondary outcomes included the individual components of the primary outcome.

The clinical composite endpoint of repeat ablation, CV, or new AAD usage as a surrogate of AF recurrence has been utilized in prior retrospective studies of large EHR databases.^24^, ^25^, ^26^ Although this clinical composite endpoint may underestimate the true rate of AF recurrence as not all patients with arrhythmia recurrence may receive treatment with additional rhythm control strategies, it is likely that patients who had a strong enough indication to warrant an initial AF ablation would have further efforts at rhythm control if they had recurrence. In addition, most patients with AF recurrence who receive additional rhythm control measures receive a combined strategy of ablation and AADs.^27^

### Statistical analysis

Included patients were stratified based on the presence of the *IL6R* Asp358Ala variant (minor C allele). Baseline characteristics, including demographics, comorbidities, and baseline medication usage, were collected for patients before their index AF ablation procedure. Comorbidities were obtained using appropriate International Classification of Diseases (ICD)-9th Revision or ICD-10th Revision codes. Two or more diagnosis codes occurring at least 30 days apart were required for the diagnosis of a comorbidity to minimize potential coding errors. Demographics, tobacco use (>100 cigarettes smoked in lifetime), and alcohol use (>2-4 drinks per month), were obtained from survey data from participants. Baseline medication use was collected for the year before the index ablation. Continuous variables were summarized with mean and SD, and categorical variables were summarized using counts and percentages. Baseline characteristics were compared between the different genotypes using chi-square tests and 2-sample t-tests for categorical variables and continuous variables, respectively. Time-to-event data were analyzed using Kaplan-Meier analysis, stratified by *IL6R* variant status, and using log-rank tests to compare the distribution of survival curves. *IL6R* variant status was modeled in a dominant fashion, comparing carriers (1 or 2 minor C alleles) vs noncarriers (no minor C allele). The index date was defined as the date of a patient’s first AF ablation procedure and patients were censored at their last clinical record. Multivariable Cox proportional hazard models were used to estimate adjusted HRs with 95% CIs for the outcomes of interest. Covariates in the multivariable model included age, female sex, time from AF diagnosis to index ablation, prior CV, prior AAD use, heart failure (HF), coronary artery disease, hypertension, diabetes, obstructive sleep apnea, chronic kidney disease, body mass index, and height. These covariates were specified a priori based on their clinical relevance and potential association with AF recurrence following ablation. In particular, prior CV, prior AAD use, and time from AF diagnosis to index ablation were included in the multivariable model, as these variables are potential surrogates of higher AF burden, which is strongly associated with increased AF recurrence. Other covariates including sex, HF, coronary artery disease, hypertension, diabetes, obstructive sleep apnea, chronic kidney disease, body mass index, and height have all been associated with AF recurrence following ablation, providing justification for their inclusion in the multivariable model.^27^, ^28^, ^29^ The proportional hazards assumption in the primary analysis was assessed using Schoenfeld residuals. All analyses were completed on the All of Us Research Workbench with Jupyter Notebook and R. Missing data were handled using multiple imputation using the *mice* package in R.

Subgroup analysis was planned to assess whether the association between *IL6R* variant status and AF recurrence was modified in patients with HF and age ≥65 years, given the generally higher rates of arrhythmia recurrence in these subgroups. Additional subgroup analyses were also performed for sex, CHA_2_DS_2_-VASc score ≥4, and race (White vs non-White) to further explore the potential heterogeneity of treatment effect across the study population.

To evaluate the robustness of the primary analysis, several sensitivity results were performed. First, we reanalyzed the effect of *IL6R* Asp358Ala on the primary endpoint using an additive (AA vs AC vs CC genotype) model to assess the potential for dose-response. Second, we repeated the analysis excluding patients without an outpatient encounter at least 30 days before their AF ablation. Third, analysis was repeated excluding patients with missing measurement data to assess the robustness of results obtained using multiple imputations. Lastly, given that ablation technologies and clinical guidelines have evolved significantly in recent years, we repeated the primary analysis using the year of the index ablation as a covariate for the multivariable model to evaluate for potential confounding from temporal improvements in ablation efficacy. To further assess the potential effect modification in patients who underwent AF ablation using more contemporary technologies, we also performed a subgroup analysis comparing the effects of the variant on AF recurrence in the subgroup of patients with their index ablation before January 1, 2020, vs afterward.

## Results

We identified 1,540 patients undergoing AF ablation with whole genome sequencing data (Figure 1). The minor *IL6R* Asp358Ala C allele frequency was 0.3763 in the cohort, of which 607 patients (39.4%) carried no copies of the *IL6R* Asp358Ala variant (AA), 707 patients (45.9%) carried 1 copy of the variant (AC), and 226 patients (14.7%) carried 2 copies of the variant (CC). Baseline characteristics of carriers vs noncarriers are shown in Table 1. Prior CV, CHA_2_DS_2_-VASc score, and baseline anticoagulation usage were higher in the noncarrier group. Prior AAD usage was common in both groups, with amiodarone representing the most commonly utilized AAD (16.4%). Beta-blocker usage was also common in the cohort, used in approximately half of the patients at baseline. The mean time from AF diagnosis to the index ablation was 1,371 days, with no significant difference between carriers and noncarriers (missing data for 223 patients in the carrier group and 98 in the noncarrier group).

**Figure 1:**
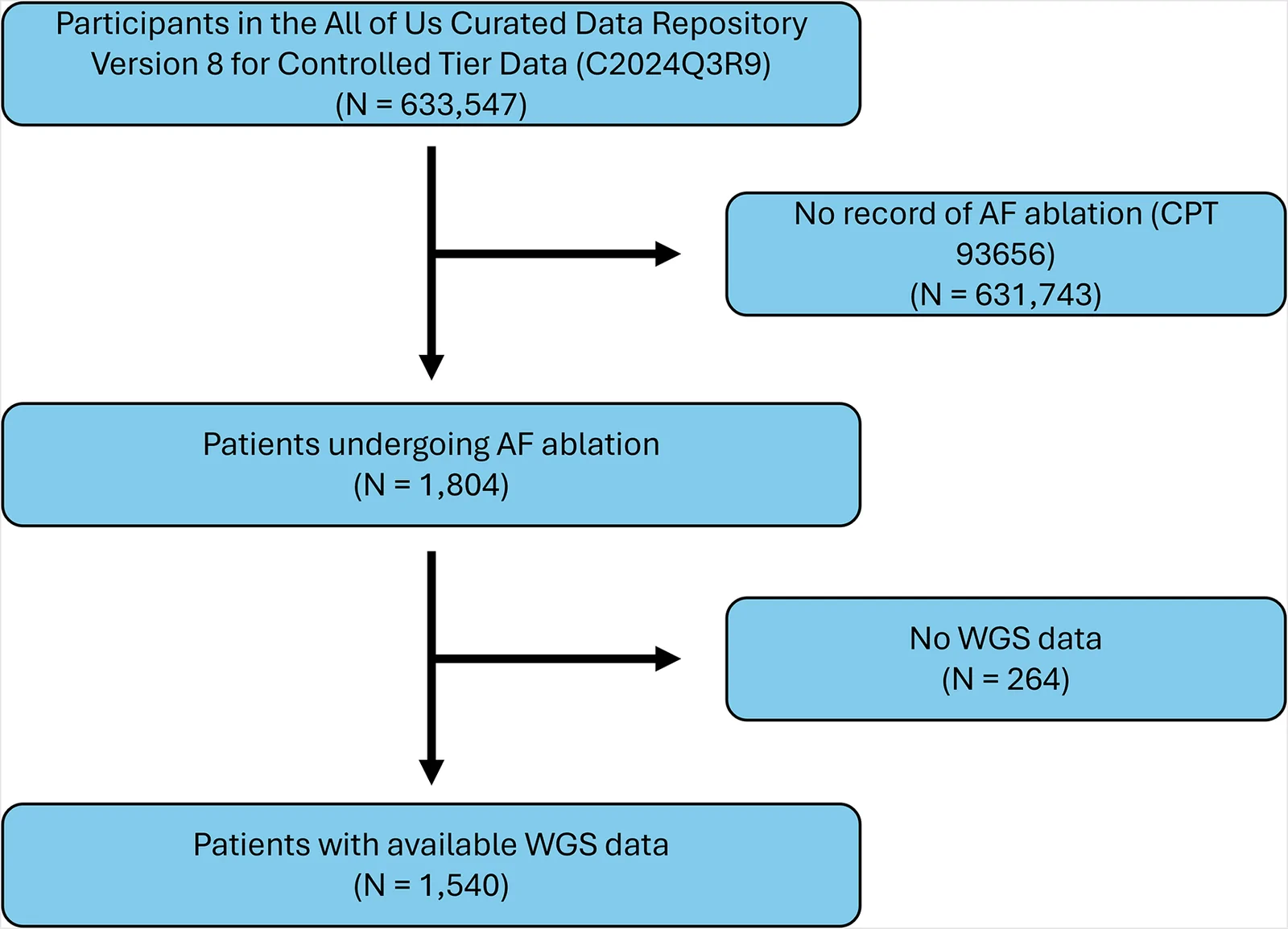
Study Flowchart Flowchart demonstrating patient selection for the cohort. Patients in the All of Us database who underwent an AF ablation procedure (identified by CPT code 93656) with available WGS data were included for analysis. AF = atrial fibrillation, WGS = whole genome sequencing; CPT = Current Procedural Terminology.

**Table 1:** Baseline Characteristics

	Overall (N = 1,540)	Non *IL6R* Asp358Ala Variant (n = 607)	*IL6R* Asp358Ala Variant (n = 933)	*P* Value
Age, y	64.2 (12.2)	64.9 (11.5)	63.7 (12.6)	0.21
Female, n	655 (42.5)	274 (45.1)	381 (40.8)	0.10
BMI, kg/m^2^	30.5 (8.8)	30.8 (11.3)	30.3 (6.8)	0.51
Height, cm	173.2 (10.9)	172.9 (10.7)	173.4 (11.0)	0.46
Tobacco use, n	726 (47.1)	293 (48.3)	433 (46.4)	0.50
Alcohol use, n	461 (29.9)	191 (31.5)	270 (28.9)	0.31
AF history				
Prior CV, n	483 (31.4)	219 (36.1)	264 (28.3)	**0.001**
Time from AF diagnosis to ablation, days	1,371.0 (1,552.1)	1,380.7 (1,493.5)	1,364.0 (1,593.8)	0.34
CHA_2_DS_2_-VASc score	2.6 (1.7)	2.7 (1.7)	2.5 (1.6)	**0.004**
Race, n				**<0.001**
White	1,213 (78.8)	461 (75.9)	752 (80.6)	
Black or African American	77 (5.00)	56 (9.2)	21 (2.3)	
Other^a^	250 (16.2)	90 (14.8)	160 (17.1)	
Baseline medication^b^, n				
Any AAD^c^	638 (41.4)	254 (41.8)	384 (41.2)	0.79
Amiodarone	253 (16.4)	106 (17.5)	147 (15.8)	
Flecainide	184 (11.9)	69 (11.4)	115 (12.3)	
Dofetilide	96 (6.2)	47 (7.7)	49 (5.3)	
Sotalol	96 (6.2)	36 (5.9)	60 (6.4)	
Dronedarone and propafenone^d^	122 (7.9)	43 (7.1)	79 (8.5)	
Beta-blocker	769 (49.9)	321 (52.9)	448 (48.0)	0.07
Calcium-channel blocker	391 (25.4)	156 (25.7)	235 (25.2)	0.89
Digoxin	83 (5.4)	28 (4.6)	55 (5.9)	0.30
Anticoagulation	912 (59.2)	386 (63.6)	526 (56.4)	**0.005**
Aspirin	416 (27.0)	175 (28.8)	241 (25.8)	0.20
ACEI/ARB	438 (28.4)	194 (32.0)	244 (26.2)	**0.015**
Loop diuretic	318 (20.6)	144 (23.7)	174 (18.7)	**0.017**
Statin	572 (37.1)	237 (39.0)	335 (35.9)	0.22
Comorbidities, n				
Hypertension	924 (60.0)	373 (61.5)	551 (59.1)	0.37
Hyperlipidemia	844 (54.8)	344 (56.7)	500 (53.6)	0.25
Diabetes	299 (19.4)	122 (20.1)	177 (19.0)	0.60
Heart failure	312 (20.3)	147 (24.2)	165 (17.7)	**0.002**
CAD	437 (28.4)	182 (30.0)	255 (27.3)	0.27
Stroke	103 (6.7)	48 (7.9)	55 (5.9)	0.14
PAD	143 (9.3)	62 (10.2)	81 (8.7)	0.32
CKD	181 (11.8)	83 (13.7)	98 (10.5)	0.06
OSA	412 (26.8)	175 (28.8)	237 (25.4)	0.14
COPD	124 (8.1)	60 (9.9)	64 (6.9)	**0.035**

Values are mean (SD) or n (%).

Significant *P* values <0.05 are represented in **bold**.

AAD = antiarrhythmic drug; ACEI = angiotension-converting enzyme inhibitor; AF = atrial fibrillation; ARB = angiotensin receptor blocker; BMI= body mass index; CAD = coronary artery disease; CKD = chronic kidney disease; COPD = chronic obstructive pulmonary disease; CV = cardioversion; IL6R = interleukin-6 receptor; OSA = obstructive sleep apnea; PAD = peripheral arterial disease.

a Includes Asian, Native Hawaiian or other Pacific Islander, American Indian or Alaska Native, Middle Eastern or North African, multiracial, did not identify with any group, and preferred not to answer. Multiple groups were consolidated due to low counts to comply with the All of Us Data and Statistics Dissemination Policy.

b Baseline medication use in the year before the index ablation.

c Patients may have been treated with multiple AAD medications before ablation; therefore, the sum of individual AADs is not equal to the count in this column.

d Dronedarone and propafenone counts were consolidated due to low counts to comply with the All of Us Data and Statistics Dissemination Policy.

The median follow-up time for the cohort was 1,317 days (IQR: 607-2064 days). The composite primary endpoint for AF recurrence (repeat ablation, CV, or new AAD therapy) occurred in 592 patients. Estimated 5- and 10-year Kaplan-Meier freedom from AF recurrence for the overall cohort was 52.8% (49.7%-56.0%) and 43.7% (39.1%-48.7%), respectively. Among patients started on AADs for arrhythmia recurrence, amiodarone was the most common AAD, utilized in 245 patients, followed by flecainide and dofetilide (n = 129 and n = 85, respectively).

There was no significant difference in AF recurrence when comparing the *IL6R* Asp358Ala variant and nonvariant groups (adjusted HR: 0.93; 95% CI: 0.79-1.10) (Figure 2A). There was also no difference in the individual components of the primary composite outcome between the groups (Table 2). Similar results were observed when analyzing AF recurrence by genotype in an additive model (AA vs AC vs CC), suggesting a lack of dose-response (Figure 2B).

**Figure 2:**
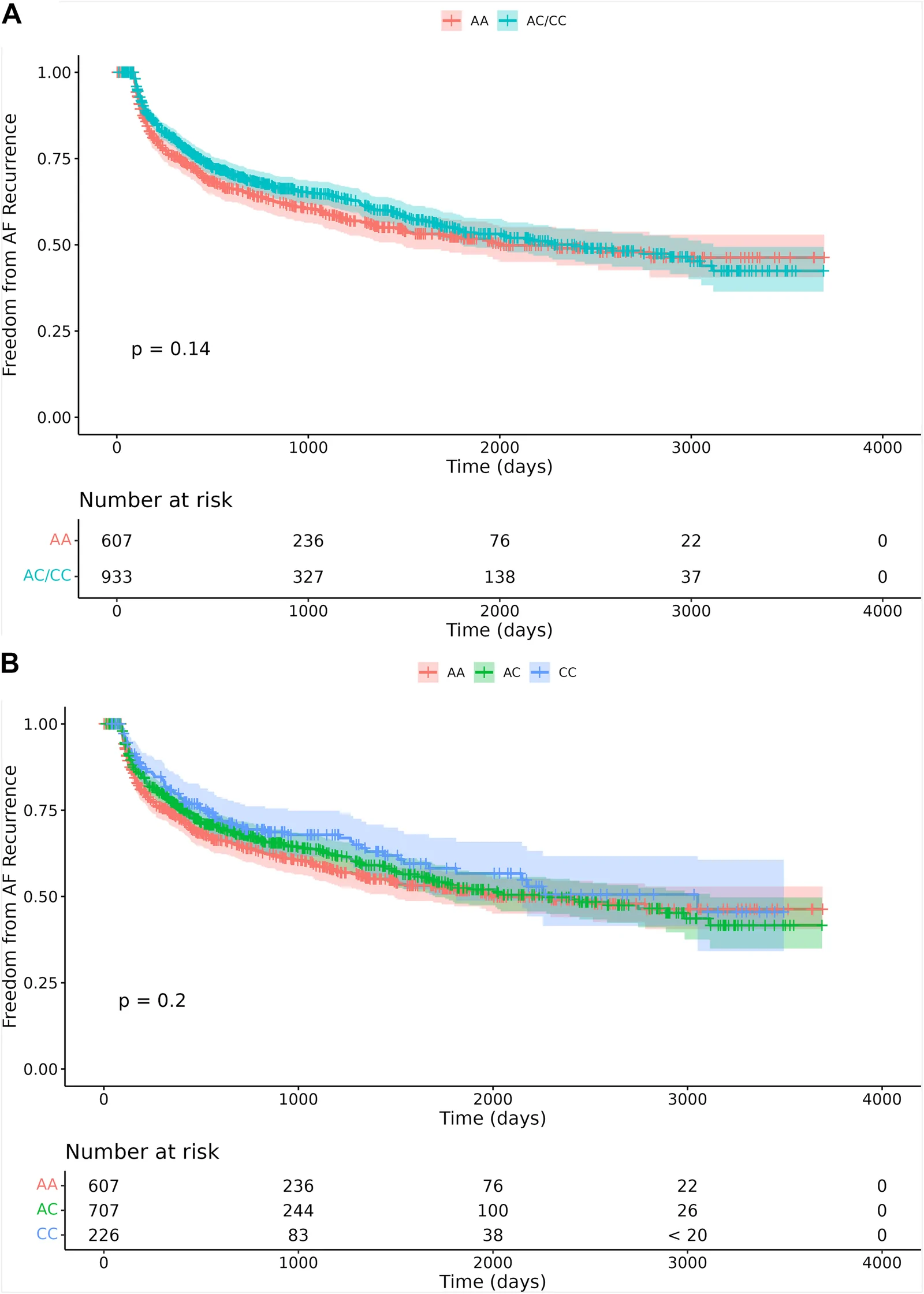
Freedom From Atrial Fibrillation Recurrence (Repeat Ablation, Cardioversion, or New Antiarrhythmic Drug Therapy) Stratified by IL6R Asp358Ala Variant Status (A) Effect of IL6R Asp358Ala using a dominant model. (B) Effect of IL6R Asp358Ala using an additive model. AC and CC genotypes represent heterozygous and homozygous carriers of the IL6R variant, respectively, while the AA genotype represents wild-type individuals (no variant alleles). Abbreviation as in Figure 1.

**Table 2:** Postablation Outcomes

	No *IL6R* Asp358Ala Variant (n = 607)^a^	*IL6R* Asp358Ala Variant (n = 933)^a^	HR (95% CI)	Adjusted HR (95% CI)^b^	*P* Value for Adjusted HR
Composite of repeat AF ablation, CV, or AAD	246 (15.9)	346 (14.2)	0.92 (0.77-1.09)	0.93 (0.79-1.10)	0.38
Repeat AF ablation	90 (4.2)	139 (4.3)	1.06 (0.80-1.41)	1.07 (0.82-1.41)	0.60
CV	106 (5.0)	144 (4.5)	0.98 (0.75-1.27)	0.99 (0.77-1.28)	0.93
AAD	200 (11.8)	281 (10.5)	0.95 (0.78-1.14)	0.93 (0.77-1.11)	0.42

Abbreviations as in Table 1.

a Data in these columns are presented as number of events (incidence rate per 100 patient-years).

b Multivariable Cox proportional hazards models adjusted for age at ablation, female sex, time from AF diagnosis to index ablation, prior CV, prior AAD use, heart failure, coronary artery disease, hypertension, diabetes, obstructive sleep apnea, chronic kidney disease, body mass index and height.

In a prespecified subgroup analysis, there was no interaction of the effect of the *IL6R* Asp358Ala variant based on age ≥65 years, sex, CHA_2_DS_2_-VASc score ≥4, or race. However, HF was seen to modify the effect of Asp358Ala on AF recurrence, with the *IL6R* variant associated with a decreased risk of AF recurrence in patients with HF (n = 312) as compared to those without HF at baseline, interaction *P* value 0.049 (Figure 3).

**Figure 3:**
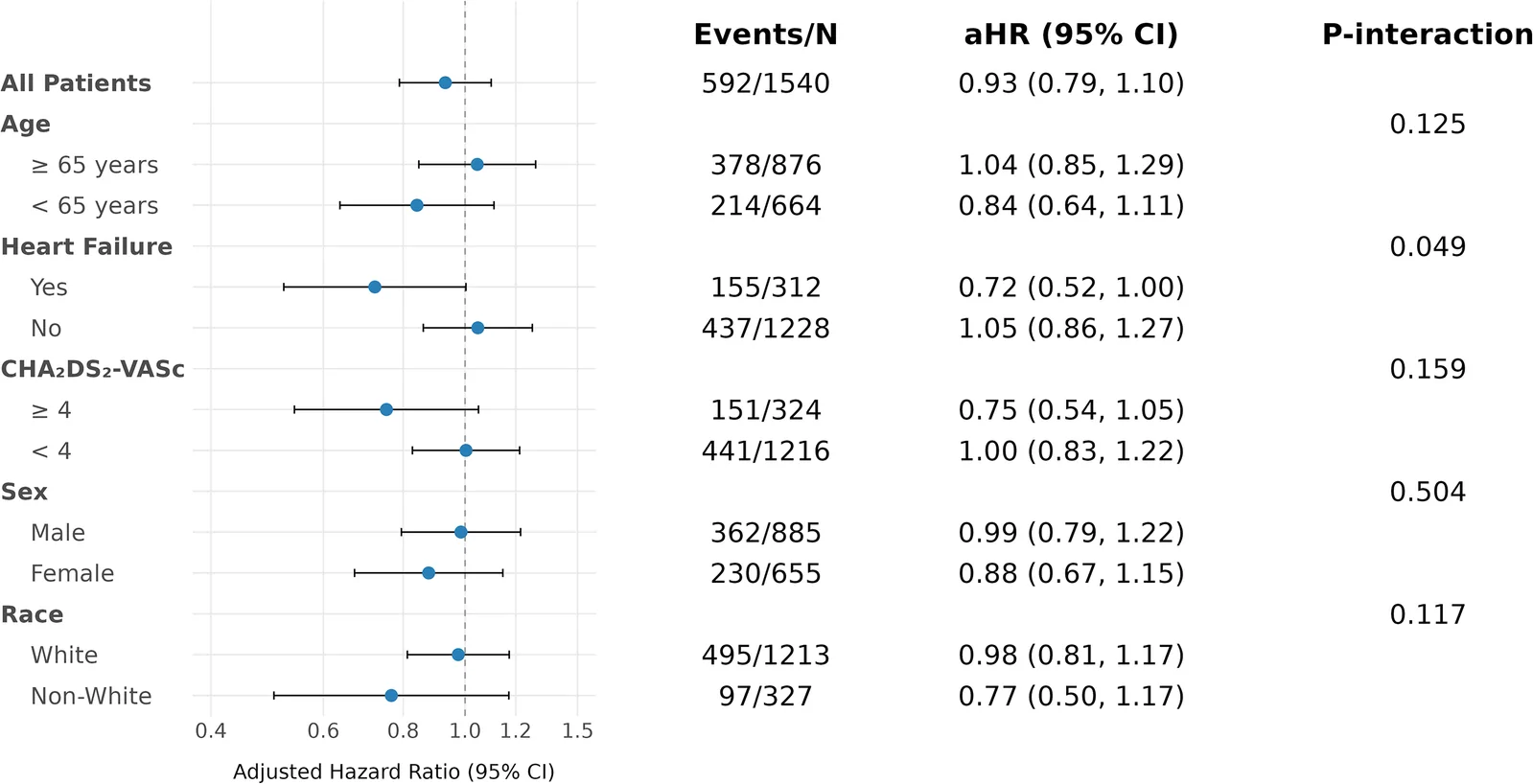
Subgroup Analysis Adjusted hazard ratio for the association of IL6R Asp358Ala variant carrier status with AF recurrence following ablation in prespecified subgroups. AF recurrence consists of the composite of repeat ablation, cardioversion, or new AAD therapy. aHR = adjusted HR.

No significant violation of the proportional hazards assumption was observed using Schoenfeld residuals. Sensitivity analyses repeating the primary analysis excluding patients without an outpatient visit encounter before their index ablation and excluding patients with missing baseline measurements yielded similar results as the primary analysis. Furthermore, sensitivity analysis repeating the primary analysis utilizing the year of the index ablation as an additional covariate in the multivariable model demonstrated no significant difference in AF recurrence between the *IL6R* Asp358Ala variant and nonvariant groups (adjusted HR: 0.93; 95% CI: 0.79-1.10). Additional subgroup analysis comparing patients who underwent their index ablation after January 1, 2020, vs before showed no effect modification based on year of the index ablation (interaction *P* = 0.23).

## Discussion

To our knowledge, the current study is the first to report on the association between genetically downregulated IL-6 signaling and long-term outcomes following AF ablation. In this large U.S.-based cohort, we observed that *IL6R* Asp358Ala carrier status, a genetic proxy for IL6R blockade, was not associated with a reduced risk of long-term AF recurrence following ablation. However, despite the neutral effects of *IL6R* Asp358Ala with regard to AF recurrence in the overall cohort, subgroup analysis identified a potential benefit from the *IL6R* variant in patients with baseline HF (Central Illustration).

**Central Illustration:**
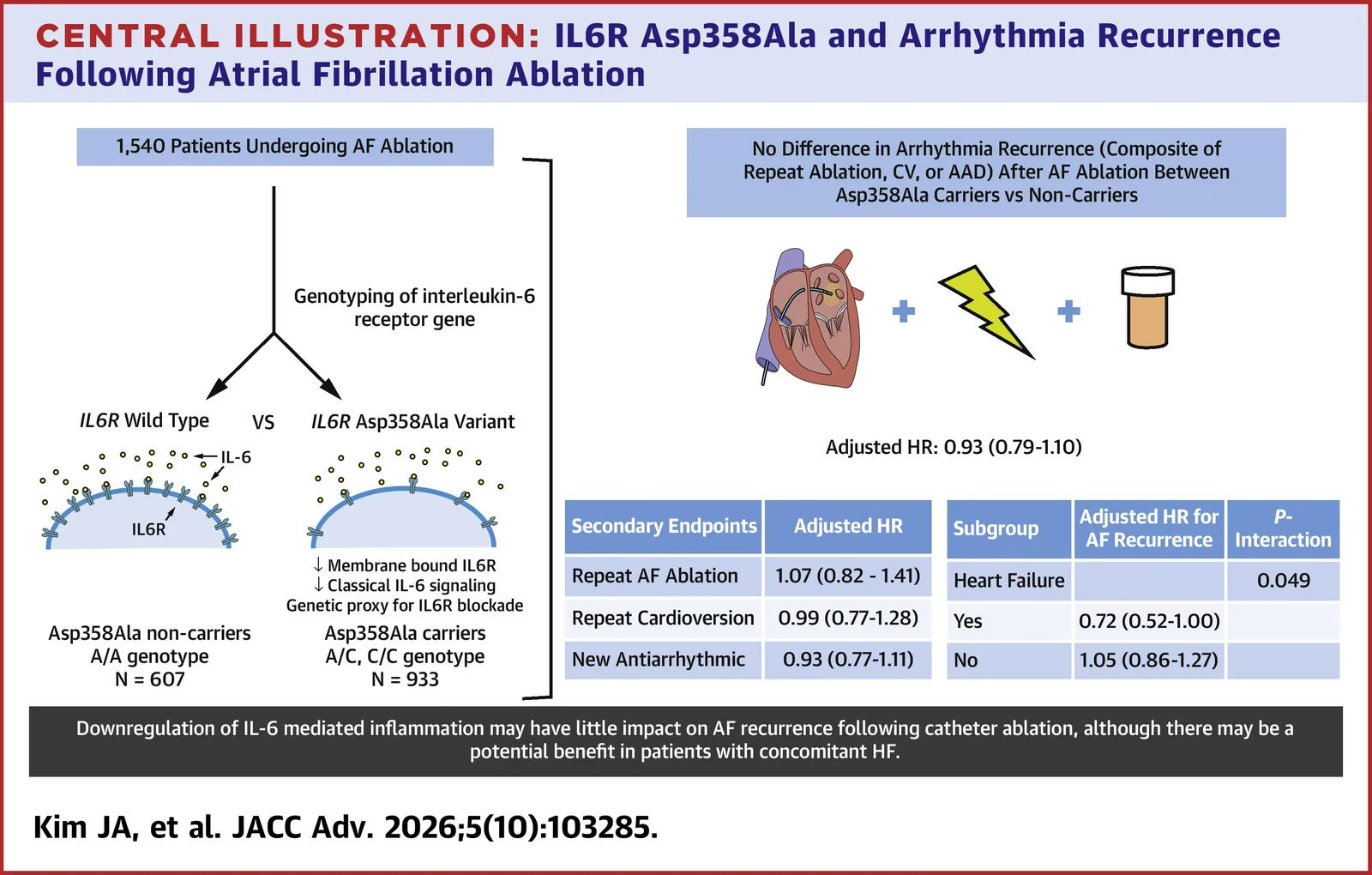
IL6R Asp358Ala and Arrhythmia Recurrence Following Atrial Fibrillation Ablation In this retrospective study of 1,540 patients undergoing a first time AF ablation in the U.S.-based All of Us Research Program, the common IL6R Asp358Ala variant, a genetic proxy for downregulated IL-6 signaling, was not associated with long-term decreased arrhythmia recurrence after ablation. However, there may be a potential benefit with the variant in patients with concomitant HF. AAD = antiarrhythmic drug; AF = atrial fibrillation; CV = cardioversion; HF = heart failure; IL = interleukin; IL6R = interleukin-6 receptor.

Inflammation is strongly linked to the development of AF, with a causal relationship demonstrated in animal models through activation of the NLRP3 (nucleotide-binding domain, leucine-rich-containing family, pyrin domain-containing-3) inflammasome and its downstream effectors, including IL-1 and IL-6.^7^^,^^30^^,^^31^ However, the precise contribution of inflammation to the overall development of AF and whether downstream effectors of the inflammatory cascade represent therapeutic targets for the treatment of AF remain unclear. Nevertheless, the feasibility of targeted immunotherapy in cardiovascular disease associated with chronic inflammation was demonstrated in the CANTOS trial, which showed a modest but statistically significant reduction in cardiovascular events with canakinumab, an IL-1β inhibitor, in patients with a previous myocardial infarction and elevated C-reactive protein (CRP).^32^ Furthermore, 1 small randomized trial of canakinumab in patients with persistent AF and elevated high-sensitivity CRP levels undergoing CV demonstrated a strong trend towards lower AF recurrence with canakinumab, although this observation did not reach statistical significance.^33^

IL-6 is consistently associated with prevalent AF and is suggested to have the strongest independent association with AF development among several inflammatory biomarkers.^8^^,^^11^, ^12^, ^13^ The IL-6 pathway is thus well-suited for evaluating the effects of attenuated inflammation on AF ablation. Despite the mechanistic link between inflammation and the development of AF,^7^^,^^8^^,^^14^ along with an association between elevated IL-6 and AF recurrence after ablation,^10^ we observed no association between the *IL6R* Asp358Ala variant and AF recurrence following ablation in our overall cohort. This finding was consistent across sensitivity analyses accounting for the year of the index ablation, indicating that the null results observed in the overall cohort are not due to confounding from temporal improvements in ablation efficacy. Altogether, this suggests that the classical IL-6 pathway may have a limited casual role in mediating long-term AF recurrence following ablation, in contrast to AF susceptibility where it has a larger impact. A previous smaller study by Wu et al^34^ reported an increase in AF recurrence following ablation with a different variant of *IL6R* (rs4845625) in a Chinese Han population. Differences in study population, consisting of predominately White patients in our study, in addition to the smaller sample size (n = 278) and shorter follow-up of only 1 year in the study by Wu et al could account for these discordant results. Furthermore, the direct effects of rs4845625, which lies in an intronic location of the *IL6R* gene, on the IL-6 pathway are less understood.

Although our findings could be potentially attributed to residual IL-6 trans-signaling with the *IL6R* Asp358Ala variant, similar findings have been observed in trials evaluating immunosuppression with periprocedural steroids or colchicine after ablation, which have not shown a clear benefit in preventing AF recurrence beyond the initial blanking period.^35^^,^^36^ As ablation fundamentally modifies the underlying atrial substrate, different mechanisms may be responsible for AF recurrence after ablation as compared to AF susceptibility, which represent distinct phenotypes.^37^ Altogether, these results suggest that IL-6 may not represent an effective therapeutic target to limit AF recurrence in the general AF population undergoing ablation. Nevertheless, these observations should be viewed as hypothesis-generating only given substantial baseline differences between carriers and noncarriers in our study and the potential for residual confounding. Furthermore, our study was inadequately powered to detect modest effects with IL-6 and AF recurrence (80% power to detect a HR ≤0.79 with α = 0.05).

Although there was no benefit with the *IL6R* Asp358Ala variant in our overall cohort, a prespecified subgroup analysis showed potential effect modification by HF status at baseline. HF and AF frequently coexist, and the presence of 1 condition can facilitate the development or progression of the other.^38^ HF is also associated with a chronic inflammatory state, as seen in the BIOSTAT-CHF cohort of HF patients, in which approximately half of all patients had elevated IL-6 levels at baseline that were associated with AF and worse clinical outcomes.^39^

The differential impact of the variant based on HF status may be attributed to distinct mechanisms underlying AF recurrence in HF vs non-HF substrates. In HF, advanced structural and electrical remodeling driven by elevated cardiac filling pressures, increased atrial fibrosis, dysregulation of intracellular calcium handling, oxidative stress, and neurohormonal activation are seen to promote AF progression and the development of arrhythmia-induced cardiomyopathy.^38^ IL-6 signaling is also associated with cardiomyocyte calcium mishandling and accelerated fibrosis, further exacerbating the arrhythmogenic substrate primed by the chronic hemodynamic and oxidative stress in HF.^40^ Given the bidirectional relationship of inflammation in both HF and AF, it is feasible that elevated IL-6 may reflect and perpetuate arrhythmic remodeling in patients whose HF is wholly or partly attributable to AF, and therefore IL-6 downregulation may suppress AF recurrence to a degree not engaged in non-HF patients. However, whether this effect on AF recurrence in HF patients is mediated more through an improvement in HF with IL-6 blockade or direct effects on AF is unclear. In addition, whether this response to IL-6 downregulation is further modified based on HF phenotype (preserved vs reduced ejection fraction and ischemic vs nonischemic) is unable to be ascertained from the current study due to limitations with ICD codes for reliably adjudicating HF phenotype in the database.

IL-6 modulation may also have a greater effect on AF recurrence in patients with an increased inflammatory status at baseline, such as those with concomitant HF, with the effects of the *IL6R* variant only reaching a therapeutic threshold when baseline inflammation is highly elevated.^39^^,^^41^ Indeed, a study of tocilizumab, a monoclonal antibody targeting IL6R, was shown to reduce the risk of AF in critically ill patients with COVID-19, a high inflammatory state.^42^ Although mechanistically plausible, our findings of interaction by HF status should be viewed cautiously given the borderline significance (*P* = 0.049), small subgroup size (n = 312), and the possibility that this represents a chance finding. Furthermore, after Bonferroni correction for multiple comparisons, the HF interaction was no longer statistically significant (Bonferroni adjusted *P* value = 0.245). The ongoing HERMES trial, evaluating the effects of ziltivekimab, a monoclonal antibody targeting IL-6, on HF outcomes in patients with HF and elevated CRP is anticipated to provide further information on the effects of targeted IL-6 therapy on AF in this high-risk population.^43^

### Study Limitations

Although our study has several strengths, which include its relatively large patient size and long-term follow-up, there are several limitations to be noted. Inherent to any study based on diagnosis codes obtained from EHR data, there is risk for miscoding and incomplete data. Furthermore, there is always the potential for residual confounding due to the retrospective, nonrandomized nature of the study. Adjustment for genetic ancestry principal components was not performed. There were also relatively few patients in our subgroup analysis for effect modification by HF status and other variables. Therefore, these results should be viewed as hypothesis-generating only and preclude any conclusions regarding causality.

Our clinical composite outcome of repeat AF ablation, CV, or new AAD therapy as a surrogate for AF recurrence may not capture the true burden of recurrence, which is typically defined using a stricter criterion of ≥30 seconds of AF in clinical trials. However, it should be noted that our estimate for freedom from arrhythmia recurrence (53% at 5 years) is strikingly similar to that seen in contemporary studies of ablation with long-term follow-up.^3^^,^^4^^,^^44^ In the CABANA trial, the 5-year rate of freedom from recurrent atrial arrhythmias following ablation, as assessed by a proprietary electrocardiogram recording monitor for symptom-activated and 24-hr auto detection, was around 50%.^44^ The concordance in our findings supports the use of our composite clinical endpoint as a suitable surrogate for arrhythmia recurrence as assessed by more traditional electrocardiogram-based monitoring methods.

The use of *IL6R* Asp358Ala as a genetic proxy for IL-6 inhibition may not fully capture the effects of more complete IL-6 inhibition with targeted biologic immunosuppressants such as tocilizumab and ziltivekimab. However, *IL6R* Asp358Ala has been commonly used as proof-of-concept for the effects of IL-6 modulation in Mendelian studies.^15^, ^16^, ^17^^,^^22^^,^^45^ We lacked granular data on AF classification (paroxysmal vs persistent vs long-standing persistent), which was not included in our analysis as ICD codes to classify AF have limited sensitivity and specificity. The lack of echocardiographic data, such as left ventricular ejection fraction and left atrial size, and inflammatory biomarker levels is also a significant limitation to our study.

Lastly, information on procedural characteristics regarding the AF ablation were unavailable. Importantly, our study did not include patients who underwent pulsed field ablation (PFA), as the All of Us data set only includes participant data up to a cutoff date of October 1, 2023, which precedes Food and Drug Administration approval of commercial PFA systems for AF ablation. Given the fundamentally distinct biophysical properties of PFA compared to thermal-based ablation technologies,^46^ it is possible that PFA may produce different results than observed in our study and is an area for further investigation.

## Conclusions

The common variant *IL6R* Asp358Ala, a genetic proxy for IL6R blockade, does not appear to influence long-term arrhythmia recurrence following AF ablation. These observations may suggest a limited role for IL-6 directed therapies in preventing AF recurrence in the general AF population undergoing ablation. However, certain patients, namely those with baseline HF, may derive potential benefit from IL-6 inhibition with regards to AF ablation success. Future prospective studies are needed to validate these findings and determine whether certain subgroups might benefit from targeted immune therapy to prevent arrhythmia recurrence after ablation.

Perspectives **COMPETENCY IN MEDICAL KNOWLEDGE:** Although chronic inflammation has been strongly linked to the development of AF, this study suggests a limited role for the IL-6 inflammatory cascade in mediating AF recurrence following catheter ablation of AF. These observations indicate that different mechanisms may underlie AF susceptibility and AF recurrence after ablation.

**TRANSLATIONAL OUTLOOK:** Immune downregulation by IL-6 antagonism, for which several biologic agents are available, may not be an effective therapy to reduce AF recurrence following ablation.

## Funding support and author disclosures

Dr Curtis serves on medical advisory boards for Janssen Pharmaceuticals, Medtronic, and Abbott; and has received honoraria for speaking from Sanofi Aventis, Abbott, and Medtronic. All other authors have reported that they have no relationships relevant to the contents of this paper to disclose.
